# Product Creativity as an Identity Issue: Through the Eyes of New Product Development Team Members

**DOI:** 10.3389/fpsyg.2021.646766

**Published:** 2021-07-14

**Authors:** Jin Suk Park, Satoko Suzuki

**Affiliations:** School of International Corporate Strategy, Hitotsubashi University Business School, Tokyo, Japan

**Keywords:** product identification, creativity, new product development, identity, novelty, meaningfulness

## Abstract

In this study, we introduce a concept of product identification that denotes the overlap between identities of a new product and its developer. As creativity is the most important identity dimension in the new product, we draw on two dimensions of creativity: novelty and meaningfulness. According to the argument that novelty represents exploration, whereas meaningfulness represents exploitation, we hypothesize that product novelty is associated with an explorative behavior of new product team members, while product meaningfulness is associated with exploitative behavior. More importantly, product identification is proposed as the mechanism that explains the amplification effect of product identity on team members. Based on survey data collected from 200 Japanese new product development (NPD) team members, we conduct a statistical analysis to test the hypotheses. The findings demonstrate the alignment between the identity of a new product and the behaviors of the NPD members, which is amplified by product identification but not by organizational identification.

## Introduction

“*What we want to do is to make a leapfrog product that is way smarter than any mobile device has ever been, and super-easy to use. This is what iPhone is.”- Steve Jobs, 2007*

In January 2007, Steve Jobs, the CEO of Apple at the time, announced the launch of the first iPhone, which would become an industry game-changer for decades. His speech accentuated the identity of Apple products as a symbol of innovation and creativity (Korschun, 2015). The employees of Apple were able to find the overlap between their self-identity and the value of creativity of the product (Ghodeswar, 2008; Gehani, 2016), which was explained with the concept of brand identification, a perception of “sameness with a particular brand” of an audience (Tuškej et al., 2013). The identification increases the engagement of employees, and eventually, the long-term success of the organization (Gehani, 2016).

In this study, we attempt to view the identification from the product level rather than from the conventional viewpoint of the organizational or brand level. A new product development (NPD) team is one of the internal stakeholder groups that is mostly involved and engaged in the focal project (Cheng and Yang, 2019; Sicotte et al., 2019); thus, NPD team members should enormously influence and be influenced by the identity embedded in a new product under development, even before the product is disclosed to the market. Among various approaches to defining the identity and value of a product or service (e.g., Al-Sabbahy et al., 2004; Burmann et al., 2009), we focus on product creativity by arguing that it is the key identity dimension of a new product. In this context of NPD literature, technological advances for innovation underscores the importance of creative behaviors of NPD team members (Ozer, 2000; Addas and Pinsonneault, 2016). Thus, the fundamental purpose of this study is to introduce the identification of internal stakeholders with product creativity and its impact on behaviors of NPD team members.

Product creativity has been defined as the composite characteristics of novelty and meaningfulness (Im and Workman, 2004; Kim et al., 2013; Han et al., 2021) in the NPD literature (e.g., Andriopoulos and Lewis, 2009; Calic and Hélie, 2018). We focus on product creativity, because it is viewed as an important construct that leads to innovation, such as a new product. Amabile et al. (1996, p. 1154) stated: “All innovation begins with creative ideas…[C]reativity by individuals and teams is a starting point for innovation.” Many efforts have been made to apply the idea of product creativity to the internal process of NPD teams and/or their parent organizations (e.g., Im et al., 2013; Kim et al., 2013); however, these contributions mainly highlight the influence of organizational factors on product creativity rather than the psychological dynamics derived from the product creativity (Greve, 2007). In other words, the knowledge of how to improve product creativity is already accumulating with the quality and quantity of research, but how the perception of product creativity of key internal stakeholders is aligned with their behaviors has not been investigated.

Filling the above research gap is critical since the strategic alignment of NPD teams with an organizational creative orientation would not be fully understood without the missing puzzle piece of an internal perception on product creativity. We introduce the concept of product identification that would be the key mechanism connecting product creativity and behaviors of NPD team members, supplementing it with the theory of organizational (Albert and Whetten, 1985; Ashforth and Mael, 1989) and brand identification (Tuškej et al., 2013; Dissanayake, 2015). As an overlap between organizational and individual identities is defined as organizational identification (Albert and Whetten, 1985; Ashforth and Mael, 1989), the similarity of self-identity with product identity can be labeled as *product identification*. We argue that once an NPD team member develops such product identification, the association between product creativity and aligned behaviors can be strengthened because of the psychological attachment to the target product.

In particular, the propositions are positive associations between product novelty and explorative behaviors of NPD team members, and between product meaningfulness and exploitative behaviors, which are moderated by product identification. The empirical test of the hypotheses offers several implications, such as a new concept of product identification that is distinct and different from organizational identification, possibility of cyclical mechanism between the new product and NPD team members, separateness of novelty and meaningfulness, and linkage with ambidexterity literature, in addition to practical contributions.

The following sections are organized in the order of (1) summarizing the theoretical background for product identification and related key concepts, such as product creativity and organizational identification, (2) building hypotheses to address the association between product creativity, product identification, and behaviors of NPD team members, (3) conducting empirical tests of the hypotheses, (4) discussing the findings with contributions and limitations of this study, and (5) concluding.

## Theoretical Background and Hypotheses

### Importance of NPD Team Members

The development of new products brings a competitive advantage to firms by creating values that cannot be easily imitated by rivals but still can be commercialized in the market (Acar et al., 2018; Zuo et al., 2019). In the information technology (IT) age today, NPD is inevitably influenced by IT (Liu and Shi, 2017). For example, an adoption of radical technologies slows down the speed of the NPD process but improves the quality of NPD innovation (Ibrahim and Obal, 2019). Standardized technology (de Vries and Verhagen, 2016; Xie et al., 2016), technology orientation (Aloulou, 2019), and preliminary technology assessment (Florén et al., 2018), all of these, improve the innovation and creativity of NPD. The current trend in research also addressed the use of artificial intelligence and its supportive function for creativity of NPD team members (Botega and da Silva, 2020).

Given the pivotal role of IT in the current decades of innovative activities of firms, such as NPD, we review innovation management literature that has provided the introductive landscape for highlighting the important role of NPD team members while reflecting the contemporary context of technology and operation. Ozer (2000) emphasized the role of the NPD team and its members as the key actors who learn, communicate, and implement technology to achieve innovation in multiple levels from individual creativity to the bottom line of a firm. The ubiquitous influences of IT NPD were summarized in several domains, such as speed (Darawong, 2021), productivity (Sicotte et al., 2019), collaboration (Su et al., 2021), communication and coordination (Moura et al., 2021), versatility, knowledge management, decision quality, and product quality (Ozer, 2000). It is not surprising that there are many recent studies attempting to connect IT and human resources management given the human aspect of NPD (e.g., Ogbeibu et al., 2020; Danabalan, 2021), which is also reflected in the creativity literature of the virtual team (Janine Viol et al., 2019).

More specifically, a subsequent study in the context of high technology (Chen et al., 2010; Marion et al., 2016) argued that creative behaviors of NPD teams brought speedy outcomes when a new and novel technology was implemented. Members using a collaborative IT tools like sharable web-storage (e.g., Google Drive) cultivated cooperative NPD team culture, leading to productive innovation. In the same vein, quality of communication using IT within an NPD team improved creativity, and finally, resulted in better performance compared with others who relied on conventional communication methods (Darawong, 2015). When it comes to versatility or multitasking of NPD team members, IT may work favorably or adversely to the creative activities of individuals depending on member-tool fits. When IT tools helped the members handling different activities simultaneously, there was a positive effect of using the tools (Ozer, 2000). Nevertheless, highly sophisticated IT tools could interrupt the productivity of individuals (Addas and Pinsonneault, 2016). To explain the mechanism of knowledge acquisition and application during an NPD process, Im et al. (2016) invited the psychological view on learning of NPD team members. For example, they argued that a balance between novelty and meaningfulness of a new product can be determined by “team members' minds” seeking a solution. Depending on those minds, the orientations of knowledge acquisition and application will be determined. Even for a better decision quality, the role of NPD team members was stressed based on the findings that the members should “have the most intimate technological knowledge about the project (Lechler and Thomas, 2015, p. 1457).” NPD team members served their pivotal roles until the final stage of NPD, ensuring product quality (Mauerhoefer et al., 2017).

In sum, all the studies on IT and NPD mentioned above indicated that team members are the most immediate actors from the beginning to the end of a development process. Gobet and Sala (2019) even suggested that the development of creative artificial intelligence should start with the understanding of human creativity. These attentions to individual members called for various approaches that would make the understanding of NPD team members more comprehensive. R and D literature even investigated politics or power games of NPD team members within firms (Kyriazis et al., 2017) with listing 39 elements for learning behaviors of NPD teams, encompassing from leadership, culture, team integration, reputation, to delegation issues (Frank et al., 2015). Next, we introduce a string of discourses on product creativity to offer another key factor linking to the important roles of the members.

### Product Creativity and Behavior of NPD Team Members

Product creativity is built upon novelty and meaningfulness (Amabile, 1983; Im and Workman, 2004; Rubera et al., 2011; Glăveanu and Beghetto, 2021). Novelty is the feature of a new product signaling its uniqueness compared with competitors in the market, while meaningfulness denotes a useful and functioning attribute of a product (Glăveanu and Beghetto, 2021). Researchers in the NPD literature have paid attention to the reception of *external* stakeholders of such novelty and meaningfulness to explain how creativity impacts organizational performance (c.f. Nakata et al., 2018). For example, Im et al. (2015) showed that when consumers perceive a new product to be very novel and/or meaningful, there were positive attitudes of consumers toward the new product. Davis et al. (2017) revealed that a similar process happens with venture investors by showing that perceived product creativity of inventors affects crowdfunding performance. However, to the knowledge of the authors, there is no study that focused on the reception of *internal* stakeholders of product creativity. Aiming to fill the gap, we focus on searching for literature that provides a theoretical backdrop for how the perceived product creativity of NPD team members affects their behavior.

Recent team identity studies (e.g., Joo et al., 2012; Oliver and Cole, 2019) are the starting point for the argument that there should be a significant overlap between product creativity and employee behaviors through an unwritten regulatory context (Gotsi et al., 2010). A regulatory context is a concept in social identity theory which argues that the internal environment of an organization, like a role expectation, signals a desirable self-identity to be shown from employees, changing their behaviors correspondingly. The perception of team members of creativity of a team can work as such pressure on and/or motivation for individuals to behave in a certain way with other team members (Voss et al., 2006). For example, the consensus of an NPD team on a culture of radical innovation significantly changed the attitudes of all team members to become more explorative (Oliver and Cole, 2019). These employees started to act as a group of rebels against traditions and bureaucracy of the company. We argue that there would be a similar mechanism between the perception of NPD team members of the creativity of the new product and their behaviors.

The advantage of relying on social identity theory is that it provides a framework to explain the psychological mechanism of how product creativity perceived by NPD team members influences their behaviors. The previous literature has emphasized the role of organizational identity in managing the strategic direction of innovation at the organizational level. For example, Tripsas and Gavetti (2000) suggested that the Japan-based MNC *Fuji* was able to change its innovation orientation by shifting its organizational identity. Fuji changed its major product line from analog (e.g., cardboard boxes) to digital (e.g., software). The innovative identity of the novel products led to a collective perception that the company was trying to explore a new market. The posture of the authors is that a new product under development also signals the elements that affect the identity of team members and, thus, their behaviors. In particular, we focus on product creativity. The behaviors of internal stakeholders (e.g., NPD teams) could change according to the signals embedded in product creativity, which reveal the strategic direction of the product.

### Product Identification

Product identification is a newly introduced mechanism in this study to elaborate the mechanism of the regulatory context and signaling. Due to the limited previous investigation on product identification, consultation with the well-established research stream on brand identity and organizational identification was required. Brand identity is a distinctive set of product attributes and qualities that can be perceived by key stakeholders, such as employees (Kirton and de Ciantis, 1986; Aaker, 1996). It can influence individual consumer identity (Keller and Richey, 2006). For example, consumers tend “to express their actual or idealized self-image” by purchasing a product with creative identity, such as iPhone and iPad. If purchasing a unique item can represent the personal identity of someone (cf. Berger and Heath, 2007), it is not surprising that a member of NPD team can find his/her identity in making the product (Abratt and Kleyn, 2012). A perceived similarity between individual and social entity, and a strong emotional bond between the two is called social identification (Park, 2014). Social identification theory adapted to the organization is known as organizational identification (Ashforth and Mael, 1989). Among various definitions and conceptualizations, a common understanding of organizational identification is that it is a status of the perception of an individual on the sameness between self-identity and the identity of an organization (Ashforth et al., 2008). For example, an employee with a strong organizational identification would say “I really feel as if this organization's problems are my own” (Allen and Meyer, 1990, p. 6). Based on a previous study on brand identity and organizational identification, we define the term *product identification* as a process and status through which an internal stakeholder finds overlap between his/her self-identity and product identity. According to the identification theory, the behavior and attitude of a person can change when there is a strong sense of similarity between an individual and a product.

The most important stakeholder of product creativity is the NPD team itself, and the attitudinal outcome of its members would be product identification at the specific level and dimension (Gotsi et al., 2010). For this study, product identification indicates the identification of NPD team members with the creativity of a new product under development. The most proximal identification mechanism for the NPD teams and their members is product identification and the distal is organizational identification. Still, the two identifications would be related. For example, Andriopoulos et al. (2018) recognized that three creative dimensions of organizational identity (i.e., guided freefall, benevolent dictatorship, and cohesive diversity) are linked to two dimensions of product creativity (i.e., novelty and meaningfulness) through an identity study on NPD team members.

### Multi-Dimensionality of Identity

**Figure 1:**
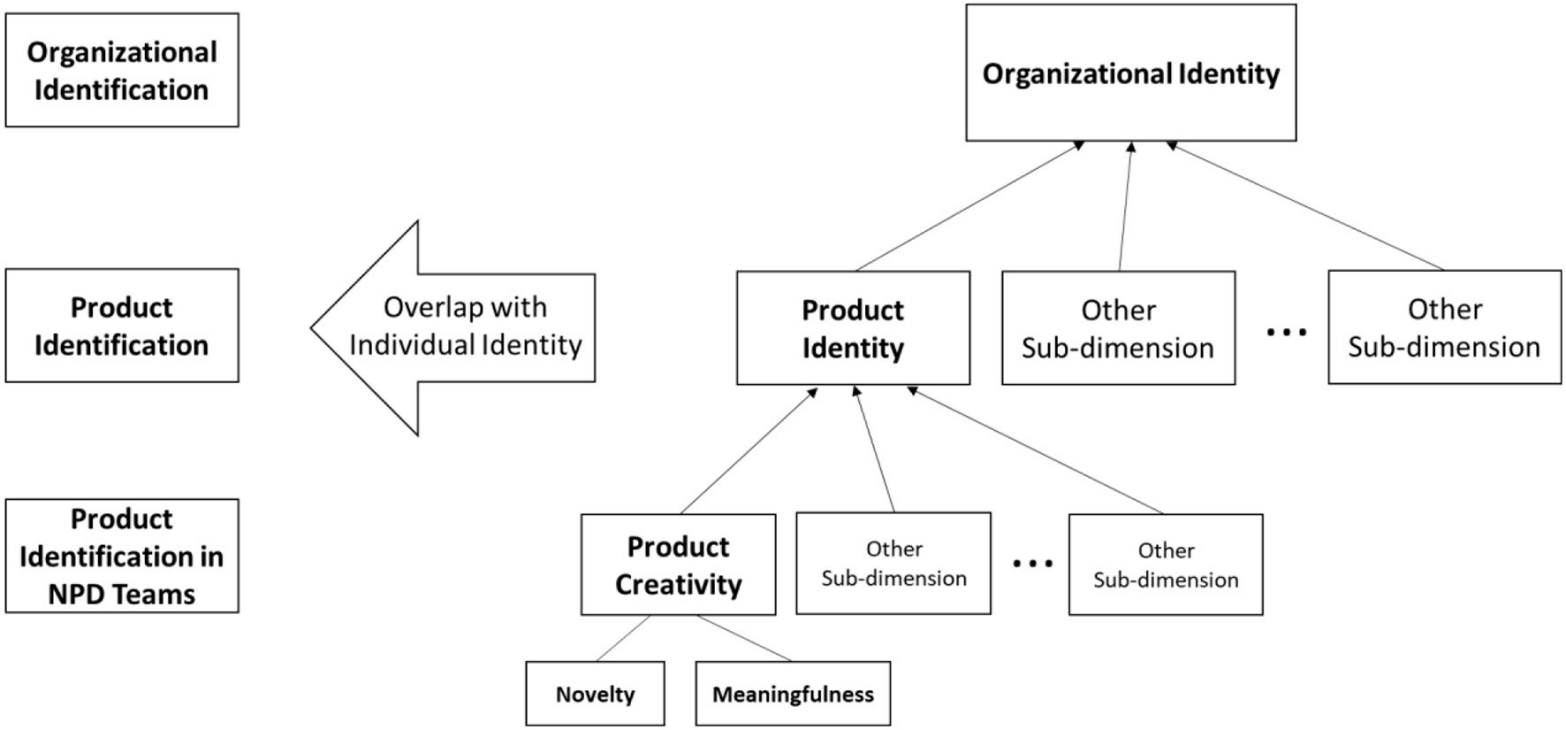
Conceptual figure of product identification.

It is also crucial to review the research stream on a multilevel and multidimensional construct of workplace identity to clarify how the two dimensions of product creativity are linked to the mindset and behaviors of NPD team members in a bigger picture of an organization (Ashforth et al., 2008; Carbonell and Escudero, 2019; Oliver and Cole, 2019). Based on the review, we presume that product identity is an overarching identity for the two sub-dimensions of product creativity, novelty, and meaningfulness, which is under the umbrella of organizational identity. In the illustration of the identity pyramid and product identification of NPD team members (Figure 1), the top of the identity pyramid is labeled as organizational identity. Then, the product identity follows as one of the dimensions of organizational identity, having its own dimensions like product creativity comprising product novelty and product meaningfulness (Andriopoulos and Lewis, 2010; Andriopoulos et al., 2018). Han et al. (2021) even used the operational definition of product creativity as a multiplicity of novelty and meaningfulness, or “Creativity (C) = Novelty (N) X Usefulness (U).”

Previous literature has revealed the multilevel nature of NPD team's creativity nested from organizational/structural level to individual/psychological level. For example, Ortiz et al. (2021) demonstrated that organizational factors such as internal social capital and absorptive capacity improved the creativity of NPD teams, which in turn had a positive effect on the bottom line of firms. Such nested creativity within an organization was also addressed with the notion of person-environment fit (Wang and Wang, 2018), suggesting that the congruency between supportive environment for creativity of an organization and creativity of employees improved the process and outcome of NPD. The study of Hui et al. (2020) on the creativity of millennials also demonstrated that the overlapped identities between self and organization led to the creativity of employees agreeing with the innovation orientation of their organization (i.e., novelty and meaningfulness). Creativity was also captured in multiple levels along with the role of team leaders, job characteristics, and organizational identification (Liu et al., 2021). Sætre and Brun (2013) insightfully interpreted such hierarchies in NPD as a continuous process of creating innovation from top to bottom, or from organization to a final product. In sum, product creativity is an important component of product identity, as well as organizational identity.

The concept of novelty is equivalent to that of *exploration* in the organizational level while meaningfulness is the product-level version of *exploitation* (Zuo et al., 2019). Exploration is a managerial system or an innovative strategy focusing on the development of new ideas by probing opportunity and ambiguity outside of an organization, whereas exploitation is maximizing the short-term return from existing resources and knowledge within an organization (March, 1991; Raisch et al., 2009). Handling these two conflicting values is considered challenging for managers and employees, and it is known as the “paradox of ambidexterity.” The NPD team members are at the front-end where they experience novelty and meaningfulness at the product level (Andriopoulos et al., 2018). The paradox can be observed by the NPD team members through product identity (Lam et al., 2015; Hameed et al., 2016). Thus, creative behaviors of NPD team members should involve separated perceptions of novelty and meaningfulness (Bonetto et al., 2021).

Empirical studies have demonstrated that product novelty and product meaningfulness can be distinguished in the eyes of NPD team members. Papachroni et al. (2016) showed in illustrative interview cases that employees can have different perceptions depending on the type of creativity:

*Innovation, is really thinking outside the box, not a day-to-day problem… [p. 1811]*

*So, to me, that's closer to my mission of innovation to explore the potential of Telco's current assets… [p. 1812]*

*It's not supposed to be reinventing the wheel, but it's duplicating it with a different notch… [p. 1812]*

*A good businessman, whether it's running a corner shop or in Telco, is always looking for new ideas, but making sure that they can run the existing business on good solid numbers… [p. 1812]*

Employees can understand the strategic intent based on the notions of product novelty and product meaningfulness, as shown in the above interview statements. The first two quotations reflect the aspect of novelty with key phrases, such as “outside the box” and “explore the potential,” whereas the last two include “duplicating it with a different notch” and “run the existing business” which reflect the aspect of meaningfulness.

By separating the two aspects of creativity, a dominant research stream in the NPD literature has revealed the causal relationship between product creativity, novelty, and meaningfulness, respectively, and subsequent changes in attitudes and/or behaviors of perceivers (e.g., Rubera et al., 2011; Im et al., 2015). In addition, the creative identity or self-view of people defining “I am creative in an explorative/exploitative way” is oftentimes malleable. It can change because of external factors (Ng and Feldman, 2012; Carlsen, 2016; van der Zanden et al., 2020). Therefore, we propose two hypotheses addressing product novelty and meaningfulness:

Hypothesis 1. There is likely to be a positive association between explorative behaviors of an NPD team member and his/her perception of product novelty.
Hypothesis 2. There is likely to be a positive association between exploitative behaviors of an NPD team member and his/her perception of product meaningfulness.

### Product Identification as a Moderator

To investigate the internal process of product creativity and its impact on the behaviors of NPD team members, we will examine the role of product identification as an amplifying factor. Figure 1 shows that product identification would occur at the lowest or the most concrete level. As discussed earlier, the definition of product identification is a perceived similarity in core characteristics between a person and a product. In this study, it is particularly important to understand the proposition of Dutton et al. (1994) that identification makes employees favorably evaluate their organizational identity and adopted activities are aligned with the perceived identity. In other words, a team member with high product identification is likely to evaluate the new product favorably, and thus aligned attitudes and/or behaviors can occur more frequently. The empirical study of Liu et al. (2021) demonstrated that organizational identification moderated the positive association between job characteristics and creativity of employees based on a positive feeling and cognitive alignment induced by organizational identification. Few studies on team identification, such as that of Hirst et al. (2009), have also indicated that team identification enhanced the motivation of members to find an aligned self with group goals. Other studies focusing on the emotional aspect (e.g., Kim and Shin, 2015) affirmed that positive emotion within a team can lead to creativity at the team level through a mechanism of improved cohesiveness. To summarize, an NPD team member with high product identification would have a cognitive and emotional attachment to the creativity of a product to make a behavioral change in line with product creativity. Thus, the following hypotheses are suggested:

Hypothesis 3. An NPD team member with higher product identification is likely to show a stronger positive association between product novelty and explorative behaviors than members with lower product identification.
Hypothesis 4. An NPD team member with higher product identification is likely to show a stronger positive association between product meaningfulness and exploitative behaviors than members with lower product identification.

The additional literature on identification was surveyed to support the hypotheses. Tang et al. (2014) showed that “team identification” facilitated knowledge-sharing within a team to improve the creativity of team members. Wang and Rode (2010) investigated that the “leader identification” of team members moderated the impact of leadership on the creativity of team members. These findings are aligned with the arguments of the authors.

## Methodology

For the purpose of this study, we consider two aspects in the context of NPD: (1) whether there is an association between product creativity and innovative behaviors of NPD team members, and (2) how product identification moderates the association between product creativity and creative activities of NPD team members. The same dataset was collected for these two separate steps. The data were collected *via* an online survey and consisted of answers from 200 respondents engaging in NPD projects in Japan collected with the help of a private research institution, and a strict screening procedure, similar to a hurdle system, was implemented: a respondent (1) must be currently employed in a for-profit organization, (2) must be a member of a NPD project, and (3) should be in the idea generation phase before the actual production of a prototype or product (Schulze and Hoegl, 2008). The sample consisted of 176 men (88%) and 24 women (12%), which is representative of the working patterns and population in Japan, especially for NPD involving R and D functions. The rank in an organization was well distributed, with 36 managers in higher positions (18%), 90 middle managers (45%), and 74 people in lower positions (37%). The average age was 52.68 years, with a minimum of 27 and a maximum of 69 (s.d. = 8.39), while years of work tenure ranged from 10 months to 45 years (mean = 19.20, s.d. = 11.81).

All 200 of the original respondents completed two sessions of the online survey at two time points, with an interval of 3 weeks to avoid common method bias (Jansen et al., 2008). As the respondents were all fluent Japanese speakers, scales in English needed to be translated into Japanese by a Japanese bilingual researcher, and they were then back-translated into English by a professional translator to ensure consistency in the items and scales (see Brislin, 1970).

### Measures

In the analysis, we included two dependent variables—explorative behavior and exploitative behavior—and four independent/moderating variables—product novelty, product meaningfulness, organizational identification, and product identification—in addition to four demographic indicators as controls. The dependent variables were measured in the first phase of the survey, while the independent and moderating variables were measured during the second phase. As the following regression analysis involves a moderating analysis, we centered the independent and moderating variables by mean value (Cohen et al., 2003).

### Explorative and Exploitative Behaviors

To measure the innovative behaviors of the NPD team members in this study, we used a self-reported 7-point Likert scale, with 1 indicating “to a very small extent” and 7 indicating “to a very large extent,” for the statements for explorative (α = 0.93) and exploitative (α = 0.95) behaviors. The scale, developed by Mom et al. (2007), included items, such as “to what extent did you, last year, engage in work-related activities that can be characterized as follows: searching for new possibilities with respect to products/services, processes or markets?” Five items applied to explorative behavior and six items to explorative behavior.

### New Product Creativity

Two aspects of the new product creativity were measured with product novelty and meaningfulness scales (Im and Workman, 2004) to match the dependent variable measures of explorative and exploitative behaviors. We followed the original scale content and structure using a 7-point Likert scale. A sample item is “the product is really out of the ordinary.” The respondents answered four items about product novelty (α = 0.87) and four items about product meaningfulness (α = 0.91). Each dimension was included in different models as the dependent variable (see **Table 2**).

### Product Identification

As product identification was a newly introduced concept, it was measured with a modified scale of organizational identification (Smidts et al., 2001). Following the previous studies that customized existing scales at a different level (e.g., Millward et al., 2007), we changed the “organization” part of the organizational identification scale to the “product” level. For example, “I feel strong ties with my company” was transformed to “I feel strong ties with the products I am developing” (α = 0.95). The items are reported in the Appendix. The organizational identification scale (Smidts et al., 2001) was also included to verify the concept of product identification.

### Control Variables

In the analysis, gender, age, team tenure, and industry were selected as control variables because of their influence on various job attitudes related to NPD (Sine et al., 2006; Mom et al., 2007; Joo et al., 2012; Carbonell and Escudero, 2019). Control variables were included in the models with the following coding: the age by years, gender as a binary variable (0 = female, 1 = male), team tenure by years, and industry by the four categories of manufacturing, retail, services, and others.

### Reliability and Validity Tests

**Figure 2:**
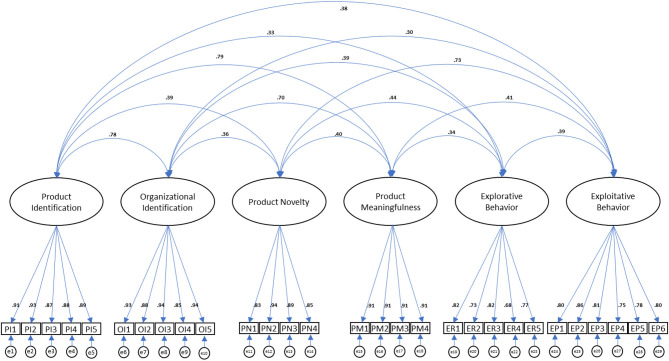
Structural equation models for reliability and validity tests.

The reliability of variables was all indicated as more than satisfactory based on Cronbach's alpha values with a minimum of 0.87. Due to the invented measures of product identification, we additionally conducted confirmatory factor analysis for a test of validity using AMOS 22 (see Figure 2). The fit indices showed that the variables used in the analysis were separated by the different dimensions (CFI = 0.94; TLI = 0.93; IFI = 0.94; RMSEA = 0.07), with a significant loading weight of every scale item for each designated construct. Although the chi-square was significant (χ^2^ [88, *N* = 200] = 157.7, *p* = 0), this statistic is extremely sensitive to sample size (Schermelleh-Engel et al., 2003; Vandenberg, 2009). The sample size is large in comparison with the suggested standard (*N* = 200 or more); thus, we can conclude that the model has a good fit overall. The average variance extracted (0.8) also indicated the high convergent validity of product identification with construct reliability (0.95), following the suggestion from Fornell and Larcker (1981).

We also assured to avoid the possibility of common method bias by including common latent variables in the CFA model (Podsakoff et al., 2003), although the data were collected in two phases separated by a 3-week interval. We found no notable risk of bias according to the indices (CMIN = 0.52, *p* = 0.472) and non-significant weights of all paths from the common variable.

## Results

To test the suggested hypotheses, we conducted a hierarchical regression analysis using the AMOS 22 software. There were two base models, Models 1 and 2, as the analysis was conducted for the two different dependent variables of explorative and exploitative behaviors. Model 2 was introduced to test H1, suggesting the main effect of product novelty on explorative behavior, while Model 3 was used to test the moderating effect of product identification (H3). Model 5 was used to test the main effect of product meaningfulness on exploitative behavior (H2). It also served as the step preceding the next hypothesis test for the interaction effect in Model 6 (H4). The descriptive statistics of all variables used in the models are shown in Table 1.

**Table 1:** Descriptive and correlation statistics.

		**Mean**	**S.D.**	**1**	**2**	**3**	**4**	**5**	**6**	**7**	**8**	**9**	**10**	**11**
1	Product novelty	0.00	1.32	1										
2	Product meaningfulness	0.00	1.24	0.401^**^	1									
3	Explorative behavior	5.36	0.85	0.443^**^	0.338^**^	1								
4	Exploitative behavior	4.99	1.05	0.727^**^	0.413^**^	0.390^**^	1							
5	Product identification	0.00	1.27	0.394^**^	0.788^**^	0.334^**^	0.377^**^	1						
6	Organizational identification	0.00	1.05	0.291^**^	0.639^**^	0.207^*^	0.349^**^	0.741^**^						
7	Gender	0.88	0.33	0.011	−0.119	−0.014	0.023	−0.057	1					
8	Age	52.68	8.39	0.050	0.131	0.030	0.114	0.172^*^	0.105	1				
9	Team tenure	19.22	11.78	0.023	−0.015	−0.110	0.042	0.030	0.087	0.423^**^	1			
10	Industry dummy 1 (manufacturing)	0.40	0.49	−0.034	−0.039	−0.042	−0.091	0.020	0.047	−0.040	0.125	1		
11	Industry dummy 2 (retail)	0.16	0.37	−0.015	0.095	0.041	0.009	0.179^*^	−0.091	0.149^*^	0.021	−0.353^**^	1	
12	Industry dummy 3 (service)	0.22	0.42	0.052	0.028	−0.024	0.086	−0.042	0.010	−0.010	−0.167^*^	−0.429^**^	**-**0.232^**^	1

* *p < 0.05*;

** *p < 0.01*.

H1 and H2 suggested the main effects of the two aspects of product creativity on the aligned behaviors of NPD team members. Table 2 shows that the results supported both hypotheses by showing significant positive coefficient values (β = 0.29, *p* < 0.01; β = 0.28, *p* < 0.05). Interestingly, there was no significant main effect of novelty on exploitative behavior (β = 0.02, n.s.) and no effect of meaningfulness on explorative behavior (β = −0.04, n.s.).

**Table 2:** Results of hierarchical regression analyses by product identification.

**Dependent variable**	**Explorative behavior**						**Exploitative behavior**					
	**Model 1**		**Model 2**		**Model 3**		**Model 4**		**Model 5**		**Model 6**	
	***B***	**S.E**.	***B***	**S.E**.	***B***	**S.E**.	***B***	**S.E**.	***B***	**S.E**.	***B***	**S.E**.
**Step 1: Control variables**												
Gender	−0.01	0.19	0.03	0.18	0.02	0.18	0.01	0.23	0.06	0.21	0.05	0.21
Age	0.09	0.01	0.06	0.01	0.04	0.01	0.11	0.01	0.03	0.01	0.02	0.01
Team tenure	−0.15	0.01	−0.14	0.01	−0.13	0.01	0.01	0.01	0.04	0.01	0.04	0.01
Industry dummy (manufacturing)	−0.05	0.16	−0.07	0.15	−0.09	0.15	−0.07	0.20	−0.10	0.18	−0.12	0.18
Industry dummy (retail)	0.00	0.20	−0.08	0.19	−0.09	0.19	−0.02	0.25	−0.08	0.23	−0.09	0.23
Industry dummy (service)	−0.07	0.18	−0.08	0.17	−0.10	0.17	0.05	0.22	0.03	0.21	0.01	0.20
**Step 2: Main effects**												
Product identification			0.20	0.07	0.26^*^	0.08			0.16	0.09	0.25^*^	0.09
Product novelty *(H1)*			0.29^**^	0.07	0.25^*^	0.07			0.02	0.08	−0.02	0.08
Product meaningfulness *(H2)*			−0.04	0.09	−0.03	0.09			0.28^*^	0.12	0.29^*^	0.11
**Step 3: Interaction**												
Product novelty* product identification *(H3)*					0.14^*^	0.03						
Product meaningfulness * product identification *(H4)*											0.19^*^	0.03
*R^2^*	0.02		0.18		0.19		0.02		0.20		0.23	
Δ*R^2^*			0.15^**^		0.02^*^				0.14^**^		0.03^*^	
*F*			12.61		16.59				15.97		19.58	

* *p < 0.05*;

** *p < 0.01*.

**Figure 3:**
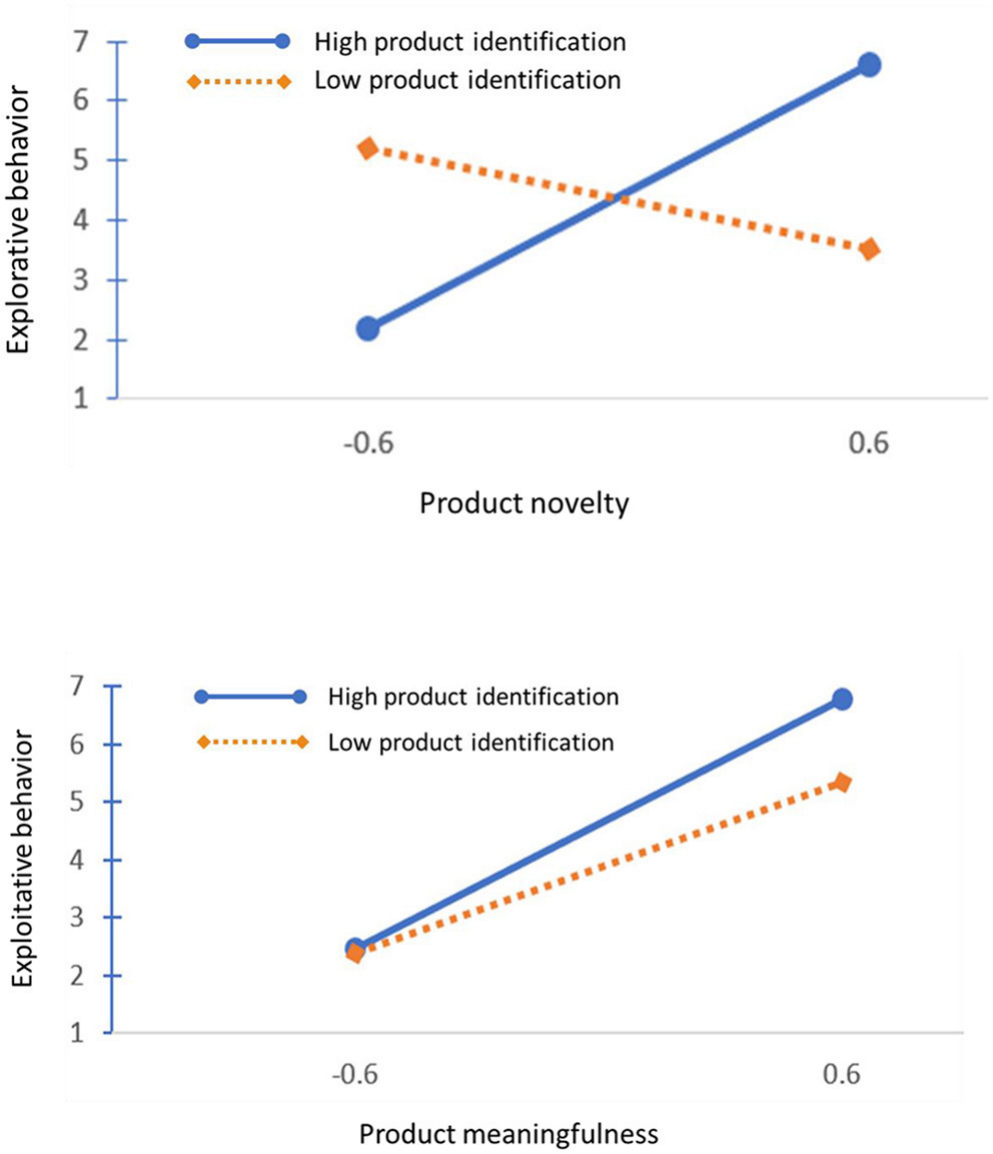
Moderating effect of product identification on the regression models.

In Models 3 and 6, the moderating effects of product identification were significant, as shown in the coefficient values of the interaction terms (β = 0.14, *p* < 0.05; β = 0.19, *p* < 0.05), supporting the predictions of H3 and H4 (see Table 2). The fits of both models significantly improved relative to Models 2 and 5, respectively (Δχ^2^ = 3.89, *p* < 0.05; Δχ^2^ = 3.61, *p* < 0.05). The overall direction of moderation was to amplify the positive main effect of product creativity on the aligned behaviors of NPD team members. The visualized patterns, however, implied that product identification might have different mechanisms for novelty and meaningfulness (see Figure 3). Product identification seems to have a strong impact on the model for product novelty by changing or removing the association between product novelty and explorative behavior. However, the result for meaningfulness demonstrated that the moderating effect was simply enhanced in the high-product-identification condition.

In the conceptual discussion earlier, we proposed that organizational identification can be at a higher level than product identification. Product identification should be discrete from organizational identification with a different psychological attitude (Millward et al., 2007). The nested relationships between the two were insinuated by a relatively high correlation (0.74, *p* < 0.01), but we focused on the most proximal mechanism, product identification. Although organizational identification was not included in the hypotheses, it would be worthwhile to empirically check if any different pattern exists compared with product identification. Hence, we conducted an additional hierarchical regression analysis by replacing product identification with organizational identification. As Table 3 shows, there was no significant effect of organizational identification that strengthens the association between product creativity and behavior of team members (β = 0.03, *n.s*.; β = 0.03, *n.s*.), and the interaction terms did not increase the fitness of the models (Δ*R*^2^ = 01, *n.s*.; Δ*R*^2^ = 0.01, *n.s*.). This implies that product identification has a distinctive mechanism despite its close tie to organizational identification. As the final step of the analysis, we examined the risk of multicollinearity along with the preventive measure of the centered variables. The estimated variance inflation factors (VIFs) of the main effects were low, with a maximum value of 3.06, while the interaction terms had a maximum value of 4.45. Both indices satisfied the suggested ceiling of 10 to exclude the risk of multicollinearity (Cohen et al., 2003).

**Table 3:** Results of hierarchical regression analyses by organizational identification.

**Dependent variable**	**Explorative behavior**						**Exploitative behavior**					
	**Model 1**		**Model 2**		**Model 3**		**Model 4**		**Model 5**		**Model 6**	
	***B***	**S.E**.	***B***	**S.E**.	***B***	**S.E**.	***B***	**S.E**.	***B***	**S.E**.	***B***	**S.E**.
**Step 1: Control variables**												
Gender	−0.01	0.19	0.03	0.18	0.02	0.18	0.01	0.23	0.06	0.21	0.05	0.21
Age	0.09	0.01	0.06	0.01	0.04	0.01	0.11	0.01	0.03	0.01	0.02	0.01
Team tenure	−0.15	0.01	−0.14	0.01	−0.13	0.01	0.01	0.01	0.04	0.01	0.04	0.01
Industry dummy (manufacturing)	−0.05	0.16	−0.07	0.15	−0.09	0.15	−0.07	0.20	−0.10	0.18	−0.12	0.18
Industry dummy (retail)	0.00	0.20	−0.08	0.19	−0.09	0.19	−0.02	0.25	−0.08	0.23	−0.09	0.23
Industry dummy (service)	−0.07	0.18	−0.08	0.17	−0.10	0.17	0.05	0.22	0.03	0.21	0.01	0.20
**Step 2: Main effects**												
Organizational identification			−0.02	0.07	−0.03	0.05			0.10	0.06	0.10	0.09
Product novelty			0.19^**^	0.07	0.19^*^	0.07			0.00	0.09	0.00	0.08
Product meaningfulness			0.09	0.08	0.11	0.08			0.27^**^	0.10	0.29^**^	0.10
**Step 3: Interaction**												
Product novelty* organizational identification					0.03	0.04						
Product meaningfulness * organizational identification											0.03	0.03
*R^2^*	0.02		0.16		0.17		0.02		0.20		0.21	
Δ*R^2^*			0.14^**^		0.01				0.14^**^		0.01	
*F*			4.14		4.12				5.36		4.88	

* *p < 0.05*;

** *p < 0.01*.

## Discussion

The primary findings of this study offer empirical evidence for the relationship between product creativity and innovative behaviors of the NPD team members, and the moderating role of product identification. When members of NPD team perceive a new product to be of high novelty (meaningfulness), their explorative (exploitative) behavior increases correspondingly, and product identification moderates the relationships.

### Theoretical Implications

The findings of this study contribute to innovation literature as we illuminate the factors that direct the innovative behaviors of NPD teams. In their influential review of innovation in marketing, Hauser et al. (2006) mentioned that understanding how firms organize for innovation is one of the important research topics. The findings show that product identification of team members affects their innovative behaviors. Although past studies showed the roles of team and expertise identification (Tang et al., 2014) and leader identification (Wang and Rode, 2010) in the innovative behaviors of team members, this study is the first research that has focused on the role of product identification. The more the NPD members identify with the products that they are working on, the more the innovative behaviors they exhibit. This focus on behaviors rather than capacities indicates the importance of alignment between strategic intention and innovative activities. Although this study is not the first one to emphasize the behavioral alignment for innovation (cf. Atuahene-Gima and Ko, 2001), it is the first seminal study to address the connection between product creativity and member creativity, along with the introduction of product identification. If the concepts of product creativity and product identification are invited to the studies on job attitudes of NPD team members, researchers could widen their theoretical approaches. For example, a mechanism explaining the effect of commitment to a NPD project on project performance (Lee and Chen, 2007) may be elaborated by product identification as well as product creativity. As the theoretical discussion has also emphasized preserving positive job attitudes while balancing structural and individual factors for an effective NPD (Lechler and Thomas, 2015), product creativity can bridge strategic orientations of two levels. Upper-level strategic intention channeled through cultural artifacts, such as office layout and language, to lower-level innovative behaviors and outcomes (Naranjo-Valencia et al., 2017; da Cruz Alves et al., 2021). The new product under development also can be considered as a sort of cultural artifact signaling the desirable directions of innovation (c.f., da Cruz Alves et al., 2021). When this bridging role of product creativity is applied to national culture, different innovative behaviors by country can be explained (Janssen et al., 2008; Shao et al., 2019), which is still an undiscovered black box in innovation research (Puente-Diaz et al., 2016b; Prim et al., 2017). By doing so, it is possible to suggest some hypotheses, such as high individualism would emphasize a novel aspect of new products to differentiate radical behaviors of the focal country from other collectivistic countries (c.f. Song and Parry, 1997).

Second, this study features product characteristics as an input of innovation (and not as an output). The previous studies in innovation literature mainly focused on the organizational and other environmental factors for innovation inputs (e.g., Burns and Stalker, 1961; Hage, 1980; Ettlie et al., 1984; Damanpour, 1991; Nemanichl et al., 2007; Bunduchi, 2009). Rosing and Zacher (2017) showed that the alignment in corporate policy, organizational resource allocation, and strategy is important to entail innovation. This study, in contrast, shows that the alignment between product creativity and innovative behaviors of internal stakeholders is important. Furthermore, this alignment may be a loop relationship between an innovator and an innovative product. In other words, the outcome of creativity (i.e., a new product) may initiate another creativity in return. Given the lexical meaning of product is a *result* of a behavior or a process (Cambridge University Press, n.d.), this is an intriguing upside-down view. When inviting the idea of affordances theory integrating self-perception on creativity and perception on environment to explain mutual influences (e.g., Piccardo, 2017), the implication suggests that the reaction of NPD team members to product creativity can generate a cyclical positive change for rich innovation. This implication runs all the way to the latest topics on open innovation. For the cases of serial entrepreneurs who continuously develop and launch new products (Yun et al., 2019), the environment of open innovation established an iterative ecosystem supporting the entrepreneurs to utilize the previous success in NPD when the next new product was launched (Ensign and Farlow, 2016). As the focus of the literature is on the economic and structural aspects of the ecosystem, the finding would shed light on the role of characteristics of a product. For example, product creativity can explain why many past and present products of serial entrepreneurs are similar in their categories from a new perspective rather than the typical rationale of financial investment (Chan et al., 2018). In other words, it would be suggested that, at least partially, features of a previous product of novelty and meaningfulness may steer the blueprints for the next product in the mind of a developer.

Third, the findings of this study contribute to the creativity and NPD literature by emphasizing the separateness of novelty and meaningfulness in new product creativity (e.g., Im and Workman, 2004; Hakala, 2011; Nakata et al., 2018). The findings showed that product novelty aligned with explorative behavior, whereas product meaningfulness aligned with exploitative behavior. However, no relationship between product novelty and exploitative behavior, as well as with product meaningfulness and explorative behavior, was found. Although NPD researchers recommend examining novelty and meaningfulness separately than combining them into a single creativity construct (Im and Workman, 2004; Nakata et al., 2018), they still consider the two characteristics together following the definition of Amabile (1983, 1988) of creativity that only something that is meaningful as well as novel can be characterized as creative. Still, the findings suggest that new products may be strong only in one aspect of product creativity. Also, this study shows that the two aspects of creativity nurture different innovative behaviors. Future research can revisit the past creativity and NPD literature and examine if there is a boundary condition for the past findings depending on the two aspects of creativity. For example, the tension between novelty and meaningfulness can be tested in sequential, alternative, and complementary relationships to understand how innovation orientation affects the creativity of NPD teams (Hakala, 2011). As technology and operation field has suggested that the standardized process of NPD would improve creativity (Acar et al., 2018), the finding adds the explanation to it by implying that the meaningfulness of product identity can easily mix with the standardized process. The study, however, reveals another side of creativity, because the regulations and standardized technology could not get along well with product novelty. This idea of separation is also well-aligned with the insight of Puente-Diaz et al. (2016a) on human perception under bipolar vs. unipolar conditions. According to their findings, two competing values that were presented simultaneously would naturally lead to a perception that status of one extreme denotes a lack of another extreme. When it is applied to this study, product with well-balanced novelty and meaningfulness, which is seemingly a bipolar condition, may have a chance to bring a paradox of creativity to NPD team members, highlighting separateness.

Fourth, this study contributes to ambidexterity literature as well. It has been proposed that organizational ambidexterity can be achieved by structural and functional balance between exploration and meaningfulness (e.g., Liu et al., 2011; Zhang and Cantwell, 2011), whereas individual ambidexterity can be led by versatile change between novel and meaningful behaviors depending on situational needs (e.g., Westergren et al., 2019). The former is called structural ambidexterity and the latter is contextual ambidexterity. This study suggests that the structural ambidexterity is applicable to contextual ambidexterity through product-level paradox. If an organization that achieved a balance between explorative and exploitative wants to implement the advantage in the level of NPD teams and members, it should be able to consider altering the focus of product identity between novelty and meaningfulness to respond to fluctuating situations. In this sense, we expect that this study will facilitate a comprehensive understanding of the dynamics of ambidexterity by linking the macroframework to the micromechanism of the perceptions and reactions of employees, which is aligned with a meso-organizational behavior approach (Molloy et al., 2010).

### Managerial Implications

There are several managerial implications of this study for the management of innovation. First, given the effects of product identification on innovative activities, managers can promote product identification through nurturing the product loyalty of NPD team members. Managers should consider the set of factors traditionally associated with brand loyalty formation, such as brand commitment (Knox and Walker, 2001; Kim et al., 2008), that may contribute to nurturing the product identification of team members.

Second, given the effects of product identity on innovative activities of NPD team members, managers should more deliberately create the image of new products. Novel yet meaningful products may be ideal; however, in reality, it may be difficult to achieve the balance as novelty and meaningfulness require different innovative activities. The three practical suggestions of Nemanichl et al. (2007) to lead NPD teams as core strategic competence are useful to articulate the practical implication of this study. They argue that to balance exploration and exploitation, managers should (1) start from the analysis on the existing structure, (2) change NPD strategy, and (3) change the behavior of leaders. Applying these three suggestions to this study, to balance product novelty and meaningfulness, executives may consider to (1) examine the current identity of new product, (2) change the product creativity, and (3) improve product identification. To generalize, managers may need to be more conscious of the type of innovation that they are working on, noting that product identity and product creativity signal strategic innovation orientation to internal stakeholders.

Third, this study employed an interdisciplinary approach encompassing the literature from both marketing and management fields. This approach may be applicable to the practice as well. For example, the internal marketing literature suggests that employees in different functions (i.e., not only marketing) play a key role in implementing marketing strategies at both strategic and tactical levels (Rafiq and Ahmed, 1993). These suggestions are now extended to the relatively newer context of “cross-functional” NPD teams (Sarin and McDermott, 2003).

## Limitations and Future Directions

Regardless of these implications, this study has limitations and room for improvement. First, all variables were collected from a self-report survey. Thus, further study can consider using archival data and/or more sophisticated analytical tools, such as hierarchical linear modeling, to improve the robustness of empirical tests. Second, as the sample covers a limited region (i.e., Japan), which has a specific business culture at the national level (Makino and Lehmberg, 2020), a generalizability issue needs to be addressed. Future research should include culturally different regions, such as North America. Third, the methodology was not able to claim the causality of association. It cannot exclude alternative explanations; and there can be reversed causality between behavioral change and perception (Carney et al., 2010). Since the focus was on the moderating mechanism of product identification, we followed the basic assumption in behavioral studies proposing that perception leads to behavior. Future research could employ an experimental method to test the causality of the identified relationships in this study. Due to the same methodological limitations, we could not delve into longitudinal variance that may affect the creativity of NPD teams. Nemanichl et al. (2007) argued that the trajectory of exploration/exploitation would change according to the different stages of an NPD process such as ideation, prototyping, and commercialization. Future study is needed to explore this issue. Fourth, we expect that the role of leadership can be elaborated during the process of interpreting product identity. A certain type of leadership may be capable of drawing the attention of followers to a balance instead of inducing a one-sided view. Further exploration of the role of leadership in the identified relationship is necessary (c.f. Hoegl and Gemuenden, 2001; Cheng and Yang, 2019; Zhang et al., 2019). For more practical studies, green product and creativity management can be new directions. Several studies already gave attention to corporate social responsibility (CSR) as an organizational identity dimension, and there was an effort to link green organizational identity to green product development (e.g., Chang et al., 2019; Ogbeibu et al., 2020). Thus, research on CSR product identity would enlighten managers leading NPD teams by adding another dimension of product identity in addition to creativity. Sports management studies are especially helpful to add more insights to such green practices at the team level. Emich et al. (2020) compared the citation network of the SCOPUS database for sport psychology and management and found that there was still an unexplored but enormous potential of synergy between the two fields. In a more general setting, Ogbeibu et al. (2020) demonstrated that human resource management focusing on green innovation led to green *team* creativity. As this study could not offer enough argument and insight on team dynamics, future study should be able to benefit from the different angle adopting team level approach. Finally, linking product identification to other individual and group level ideas, such as creative efficacy (Tierney and Farmer, 2002), is also a prospective venue for further investigation.

## Conclusion

This study explicitly addressed product identity that confronts NPD team members with a creativity paradox. Product identity was discussed as a parent category of product creativity that would be perceived in two distinctive dimensions of novelty and meaningfulness. The empirical test revealed that there was product identification moderating the positive association between product novelty and explorative behaviors as well as between product meaningfulness and exploitative behaviors. The existence of product identification was reaffirmed by its idiosyncratic mechanism compared with organizational identification. Thus, the creativity of NPD teams and their members can be improved by well-directed product identity. The importance of the perception of internal audiences on the new product under development was accentuated.

## Data Availability Statement

The original contributions presented in the study are included in the article/supplementary material, further inquiries can be directed to the corresponding author/s.

## Author Contributions

JP: data analysis, writing the draft, revision, and other submission processes. SS: data collection and analysis, critical reviews and suggestions, revision, and other submission processes. All authors contributed to the article and approved the submitted version.

## Conflict of Interest

The authors declare that the research was conducted in the absence of any commercial or financial relationships that could be construed as a potential conflict of interest.

## Appendix

**Product identification measurement and organizational identification measurement**

A. Product identification: New.
I feel strong ties with the products I am developing.
I experience a strong sense of attachment to the products I am developing.
I feel proud to work on the products I am developing.
I am sufficiently acknowledged in the new product development team.
I am glad to be a part of developing the products.
B. Organizational identification: Smidts et al. (2001).
I feel strong ties with my company.
I experience a strong sense of belonging to my company
I feel proud to work for my company.
I am sufficiently acknowledged in my company.
I am glad to be a member of my company.
